# Supplementary material for: Incorporation of Suppression of Tumorigenicity 2 into Random Survival Forests for Enhancing Prediction of Short-Term Prognosis in Community-ACQUIRED Pneumonia
Source: J Clin Med. 2022 Oct 12;11(20):6015. doi: 10.3390/jcm11206015 (PMC9605170; doi:10.3390/jcm11206015)
Supplement: Supplementary file 1 [file jcm-11-06015-s001.zip › jcm-1835308-supplementary.pdf]

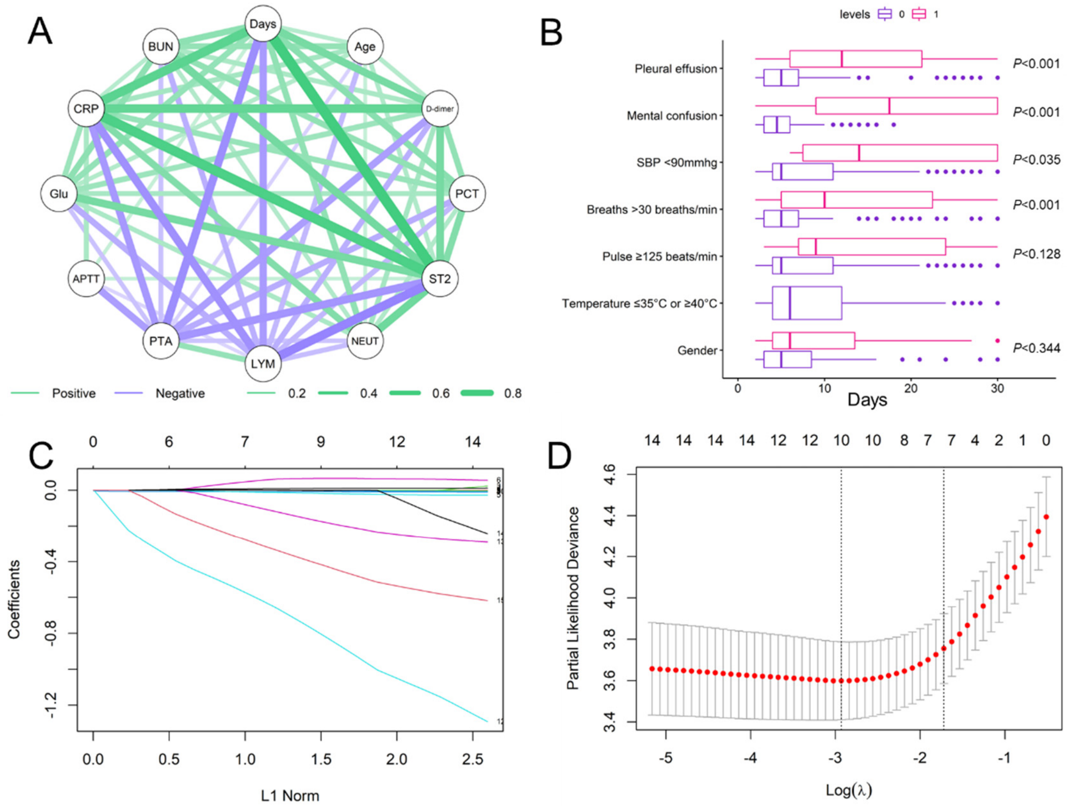

Figure S1. Feature selection of the prediction model. (**A**) The correlation network of selected variables as the variables correlated with clinical stability. (**B**) The time to clinical stability in different groups. (**C**) Lasso regression of the selected 15 variables. (**D**) The model performs best when the number of variables was 10. Days: days to clinical stability, PCT: Procalcitonin, NEUT: Neutrophils, LYM: Lymphocyte, PTA: Prothrombin activity, APTT: Activated partial thromboplastin time, Glu: glucose, CRP: C-reactive protein, BUN: Blood urea nitrogen.
